# Thermally Activated Delayed Fluorescence in Neutral and Cationic Copper(I) Complexes with the 2-(4-Thiazolyl)benzimidazole Ligand

**DOI:** 10.1021/acs.inorgchem.3c01409

**Published:** 2023-06-22

**Authors:** Adrián Alconchel, Olga Crespo, M. Concepción Gimeno

**Affiliations:** Departamento de Química Inorgánica, Instituto de Síntesis Química y Catálisis Homogénea (ISQCH), Universidad de Zaragoza-CSIC, E-50009 Zaragoza, Spain

## Abstract

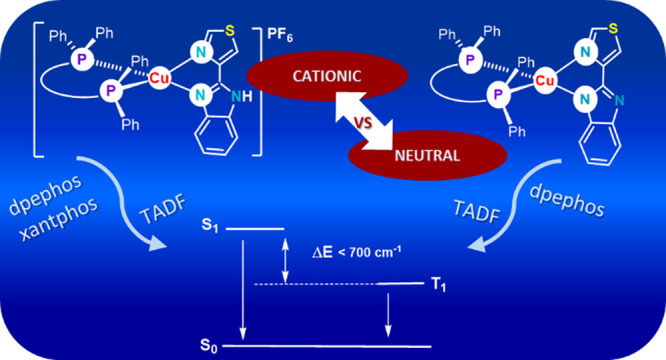

Cationic [Cu(P^P)(Htbz)]PF_6_ [P^P = xantphos, dpephos; Htbz = 2-(4-thiazolyl)benzimidazole] and the corresponding neutral complexes [Cu(P^P)(tbz)], obtained through deprotonation of the diimine ligand, have been synthesized with the aim of analyzing the role of the diphosphane and Htbz deprotonation in the emissive properties of these complexes. For the study of the diphosphane effect, the luminescence properties of these compounds have been compared with those of the reported analogous derivatives with Htbz and carborane diphosphanes. Complexes [Cu(P^P)(Htbz)]PF_6_ (P^P = xantphos, dpephos) and [Cu(dpephos)(tbz)] display thermally activated delayed fluorescence, which has been studied, revealing a Δ*E*(S_1_–T_1_) between 658 and 455 cm^–1^. Theoretical calculations indicate different origins for the absorptions, leading to the observed emissions.

## Abstract 2

This work studies the influence of the diphosphane and the charge in the emissive properties of complexes [Cu(P^P)(Htbz)]PF_6_, Htbz = 2-(4-thiazolyl)benzimidazole] and the corresponding neutral analogous [Cu(P^P)(tbz)], obtained by deprotonation of the diimine ligand. TADF has been found for the blue or green emitters [Cu(P^P)(Htbz)]PF_6_ and [Cu(dpephos)(tbz)] with Δ*E*(S_1_−T_1_) values between 658 and 455 cm^−1^. Theoretical calculations reveal a different origin for the absorptions, leading to the observed emissions.

## Introduction

**Figure 1:**
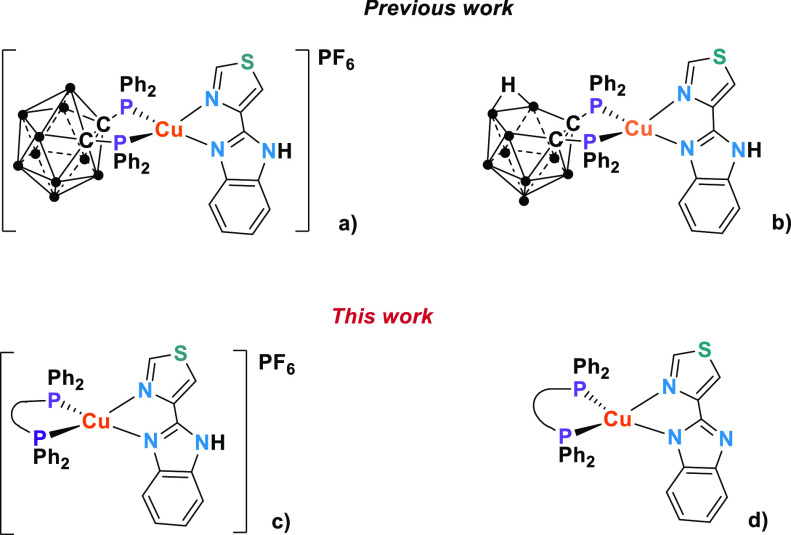
Previous work: complexes with carborane diphosphanes (• = BH). (a) Cationic complex with the *closo*-diphosphane dppcc, (b) neutral complex with the *nido*-diphosphane dppnc. This work: Heteroleptic complexes with xantphos and dpephos. (c) Cationic complexes with the Htbz diimine, and (d) neutral complexes with the tbz^–^ diimine.

Thermally activated delayed fluorescence (TADF) has been reported for many copper(I) emissive complexes, which make these species attractive candidates for OLEDs and solar cell design. This fact and the low cost and wider availability of copper, compared to those of the mainly used metals Pt and Ir, have led to growing interest in copper complexes as part of the emissive layer of OLEDs. Recent reviews on [Cu(P^P)(N^N)]^*n*^ compounds (*n* = 0, 1; P^P = diphosphane, N^N = diimine) resume their emissive properties, try to rationalize the influence of the ligands, and/or analyze their suitability for light emitting devices.^1−4^ Despite the diversity of the studies resumed in these reviews, the number of reports on [Cu(P^P)(N^N)]^*n*^ complexes is growing, demonstrating that modulation of the color, quantum yield, stability, and other properties of these systems still represents a hot topic in this field.^5−12^ Among the studies on [Cu(P^P)(N^N)]^*n*^ complexes, those concerning the analysis of the effect of the diphosphane are scarcely represented.^13−16^ In this sense, we have recently reported on the analysis of the influence of the *closo-* or *nido-*nature^17^ of a carborane diphosphane in the emissive properties of cationic or neutral copper complexes, respectively, with the 2-(4-thiazolyl)benzimidazole (Htbz) diimine (Figure 1) and compared their properties with those of previously reported copper compounds with the same *nido*-diphosphane and other diimine ligands.^18,19^

Our aim in this work is to extend the study of the analysis of the diphosphane effect in complexes with the Htbz ligand by replacing the carborane diphosphane by no-carborane diphosphanes in complexes of stoichiometry [Cu(P^P)(Htbz)]^+^. Li and co-workers in 2009 and Dansong in 2010 have reported the emissive properties of compound [Cu(dpephos)(Htbz)]BF_4_, both in dichloromethane solution and PMMA film,^20,21^ and the effect of substitution of the NH hydrogen atom of the Htbz ligand by Et or 4-carbazoylbutyl groups. We analyze the emissive properties in the solid state of [Cu(dpephos)(Htbz)]PF_6_.

Another important objective of this work is to explore the consequences in the luminescence behavior of the deprotonation of the Htbz ligand, leading to neutral complexes.

Thus, in this work, we present the emissive properties in the solid state of [Cu(P^P)(Htbz)]PF_6_ [P^P = xantphos, dpephos) and the corresponding neutral complexes obtained by deprotonation of the diimine ligand, [Cu(P^P)(tbz)] (Figure 1) with the aim of understanding both the influence of the diphosphane and the diimine deprotonation in the emissive properties of complexes.

## Discussion

### Synthesis and Characterization

**Scheme 1:**
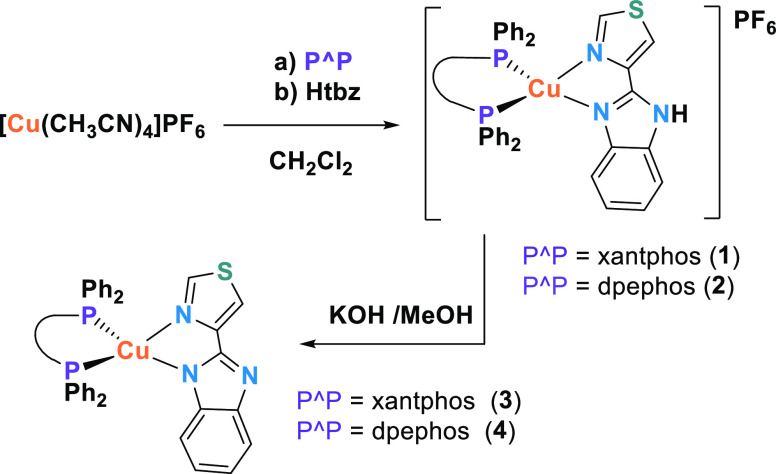
Synthetic Procedures for **1**–**4**

Reaction of [Cu(CH_3_CN)_4_]PF_6_ with the corresponding diphosphane and further addition of 2-(4-thiazolyl)benzimidazole (Htbz) affords the cationic complexes [Cu(P^P)(Htbz)]PF_6_ (P^P = xantphos (**1**), dpephos (**2**)]. Further treatment of these cationic complexes with KOH in methanol leads to the neutral compounds [Cu(P^P)(tbz)] (P^P = xantphos (**3**), dpephos (**4**)] (Scheme 1).

The resonance corresponding to the equivalent phosphorus atoms is observed in the ^31^P{^1^H} NMR spectra of complexes **1**–**4** between −13 and −16 ppm. In their ^1^H NMR spectra, some of the resonances corresponding to xantphos or dpephos overlap with those of the Htbz or tbz^–^ ligands (see the experimental part). The NH hydrogen atom of the Htbz ligand is not present in the spectra of **3** and **4** and appears at about 12 ppm in those of **1** and **2**.

**Figure 2:**
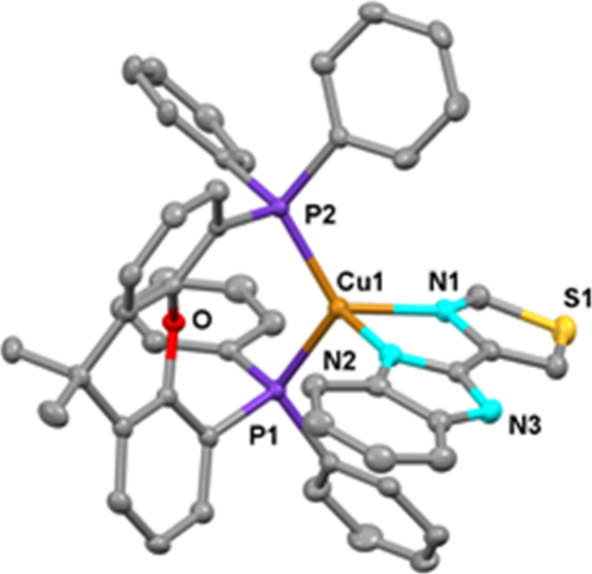
Ortep diagram of the cation of compound **1**. Ellipsoids represent 50% probability. Hydrogen atoms are not shown to improve clarity. Bond distances (Å) and angles (°): Cu1–N1 2.127(3), Cu1–N2 2.076(3), Cu1–P1 2.2588(9), Cu1–P2 2.2374(8), N1–Cu1–N2 80.531(10), and P1–Cu–P2 116.98(3).

**Figure 3:**
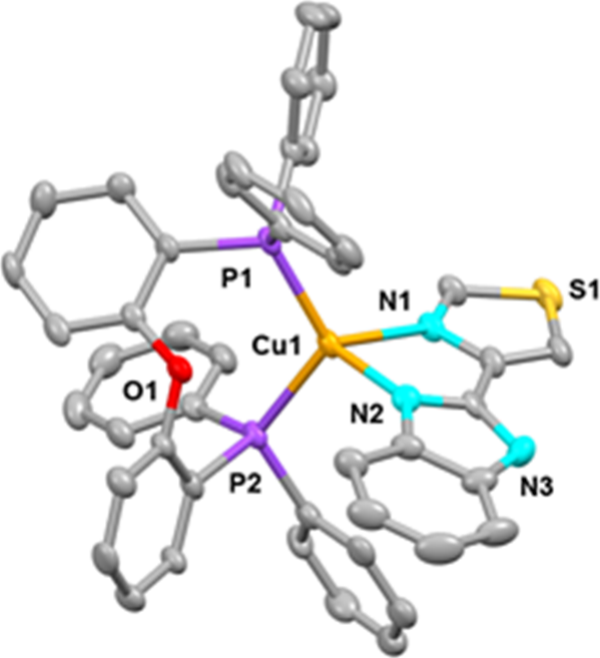
Ortep diagram of the cation of compound **2**. Ellipsoids represent 50% probability. Hydrogen atoms are not shown to improve clarity. Bond distances (Å) and angles (°): Cu1–N1 2.153(6), Cu1–N2 2.059(6), Cu1–P1 2.244(2), Cu1–P2 2.271(2), N2–Cu1–N1 79.2(2), and P1–Cu–P2 116.76(7).

**Figure 4:**
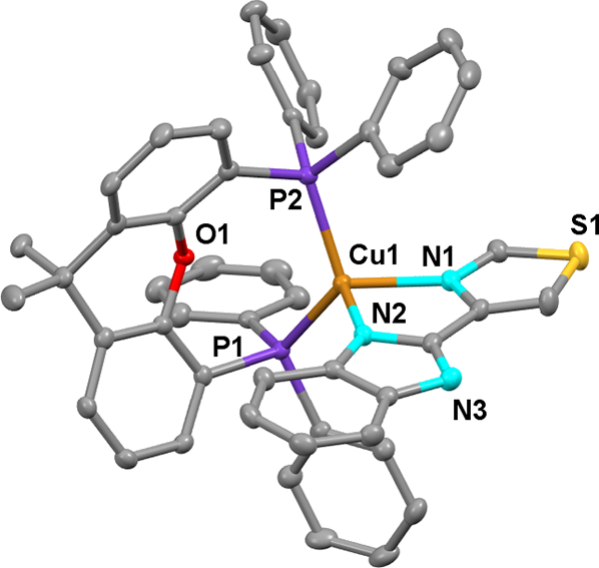
Ortep diagram of compound **3**. Ellipsoids represent 50% probability. Hydrogen atoms are not shown to improve clarity. Bond distances (Å) and angles (°): Cu1–N1 2.1014(18), Cu1–N2 2.0290(19), Cu1–P1 2.2230(7), Cu1–P2 2.2446(7), N1–Cu1–N2 82.06(8), P1–Cu–P2 120.02(2).

**Figure 5:**
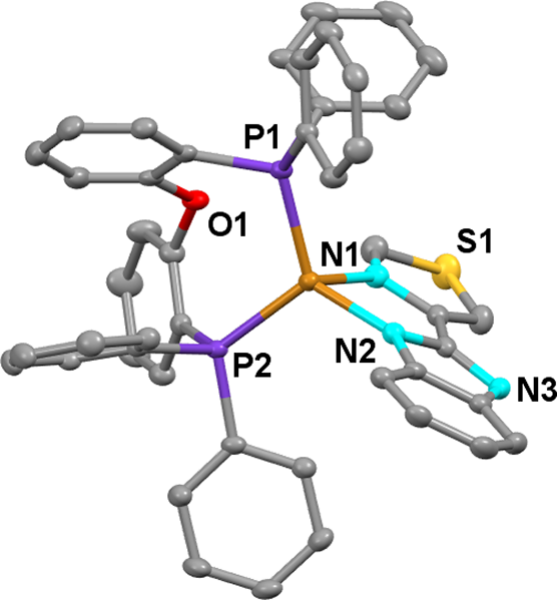
Ortep diagram of compound **4**. Ellipsoids represent 50% probability. Hydrogen atoms are not shown to improve clarity. Bond distances (Å) and angles (°): Cu1–N1 2.1372(15), Cu1–N2 2.0026(15), Cu1–P1 2.2289(5), Cu1–P2 2.2580(5), N1–Cu1–N2 80.63(19), P1–Cu–P2 115.068(6).

The crystal structures of complexes **1**–**4** have been elucidated by X-ray crystal diffraction studies (Figures 2–5). The N–Cu–N and P–Cu–P bite angles are around 80° and 120°, respectively. Thus, the distortion from the ideal tetrahedral geometry seems to be mainly induced by the narrow bite angle of the diimine. Small differences may be observed when comparing analogous complexes with the Htbz and tbz^–^. In general, Cu–N and Cu–P bond distances are shorter for the neutral complexes (**3** and **4**) than for the corresponding cationic species (**1** and **2**).

The angle between the P1–Cu1–P2 and N1–Cu1–N2 planes (α) is related with the distortion from the ideal tetrahedral geometry. The minor distortion is observed for **4** (α = 89.23°), the corresponding values for **1**, **2**, and **3** are 82.86°, 86.93°, and 84.15°, respectively.

Thermogravimetric analysis of complexes **1**–**4** (ESI) shows their stabilities below 300 °C. These thermal stabilities compare well with those reported for complexes [Cu(dpephos)(N^N)]^+^ (N^N = Htbz or substituted Htbz)^20,21^ and also for compounds [Cu(P^∧^P)(dpa)]^+^ (dpa = 2,2′-dipyridylamine; P^∧^P = dpephos, Binap or 2 PPh_3_).^22^ For the latter [Cu(P^∧^P)(dpa)]^+^ species, similar profiles to that observed for compounds **1**–**4** have been reported: two main step weigh losses are described, with the first one pointing out to the loss of the diphosphane.

### Luminescence Properties and Theoretical Studies

Complexes **1**, **2**, and **4** are emissive in the solid state, both at room temperature and 77 K (Table 1), whereas complex **3** is only luminescent at 77 K. For all the complexes, a great increment of the lifetime is observed at 77 K, compared with that at room temperature, this increment is specially marked for **4**. Structured bands are observed at 77 K. Emission energy of **2** in the solid state is consistent with that reported for [Cu(dpephos)(Htbz)]BF_4_ at room temperature in dichloromethane solution (515 nm) and as PMMA film (525 nm).^20,21^ Lifetime in the solid state at room temperature for **2** is longer (40 μs) than those reported in dichloromethane solution (7.2 μs, biexponential decay pattern) and PMMA film (7 μs) for [Cu(dpephos)(Htbz)]BF_4_. Quantum yield of **2** is in between that reported in dichloromethane solution (0.2%) and PMMA film (15%) for [Cu(dpephos)(Htbz)]BF_4_.

**Table 1:** Emissive Properties of Complexes **1–4** as Powder Samples

	λ_em_^a^ (nm)	λ_ex_^a^ (nm)	Φ^b^ (%)	τ^c^ (ms)
**1** R. T.	515	332, 400	1	0.029 (0.997)
77 K	505	325, 370		3.395 (0.997)
**2** R. T.	520	400	1	0.040 (0.995)
77 K	510	326, 385		3.516 (0.999)
**3** 77 K	484	330		20.13 (0.993)
**4** R. T.	495	365	3	0.757 (0.995)
77 K	492	370		19.15 (0.997)

a λ_em_: emission maxima, λ_ex_: excitation maxima.

b Quantum yield (Φ), measured at λ_ex_ 360 nm (**1**, **4**) or 400 nm (**2**).

c Lifetime (τ) (Chi-Square). Measurements have been carried out using maxima excitation and emission wavelength (see the Supporting Information).

**Figure 6:**
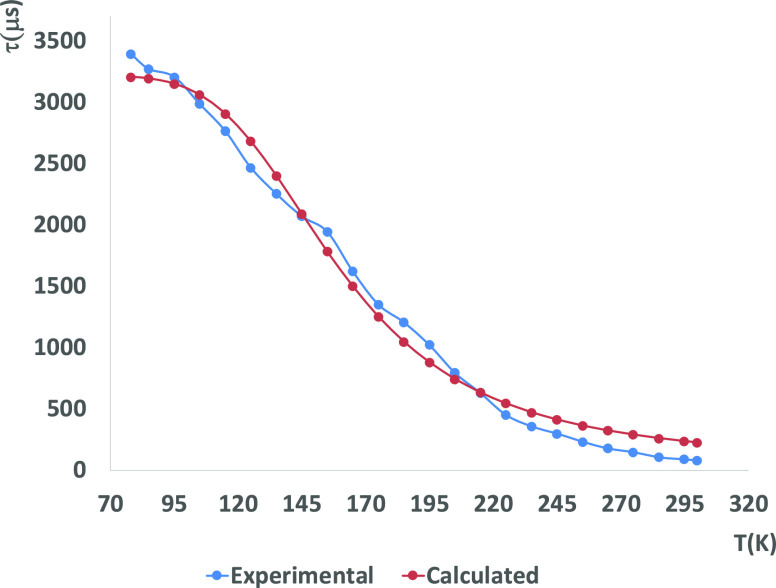
Temperature dependence of the emission lifetime of complex **1** in the solid state with fitting values using eq 1.

**Figure 7:**
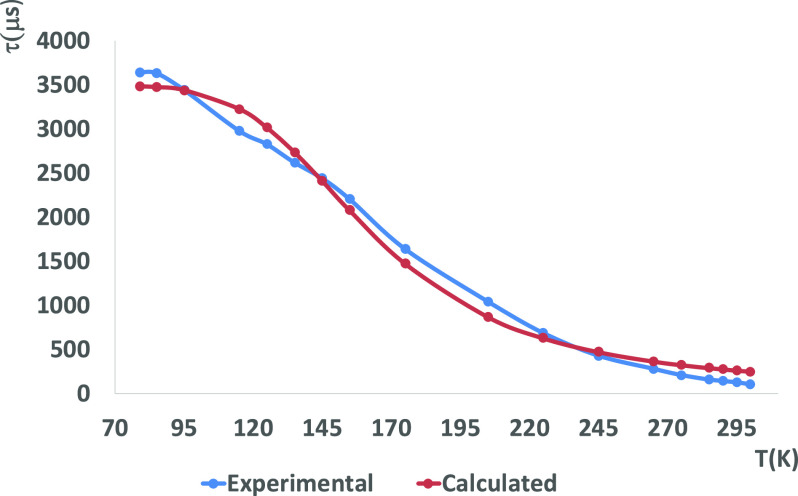
Temperature dependence of the emission lifetime of complex **2** in the solid state with fitting values using eq 1.

**Figure 8:**
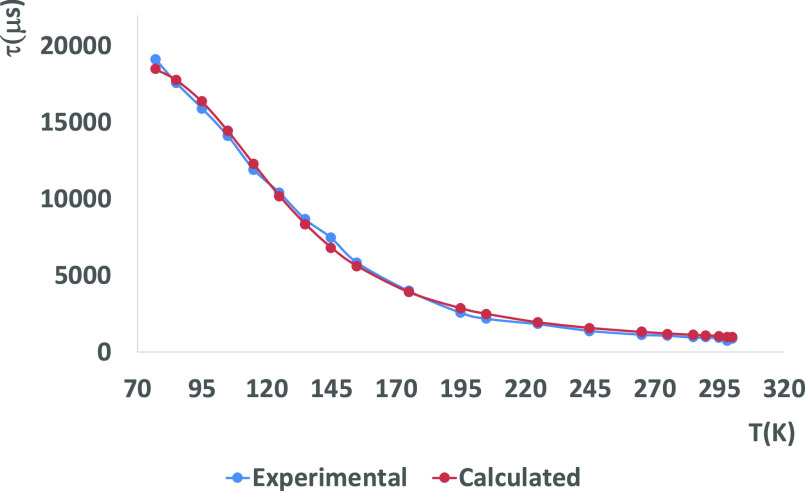
Temperature dependence of the emission lifetime of complex **4** in the solid state with fitting values using eq 1.

As commented above, Table 1 shows an important increment of the lifetimes upon cooling for **1**, **2**, and **4**, which is specially marked for **4**. The observation of longer lifetimes and a red shift of the emission upon cooling has been argued to propose the presence of TADF.^23^ Complexes **1–4** display broad emission bands, and at 77 K, these emission bands exhibit a vibronic structure. These two facts may mask a red shift. Thus, in order to prove the presence of TADF, the modification of the lifetime with the temperature has been studied for complexes **1**, **2**, and **4** (Figures 6, 7, and 8). Lifetime values of the first singlet and triplet excited sates [τ(S_1_) and τ(T_1_), respectively] and the energy gap between the first singlet and triplet excited states [Δ*E*(S_1_–T_1_)] may be calculated by fitting the experimental lifetime values at different temperatures to eq 1 (*K*_B_ = Boltzman constant) using the least-squares fitting method.
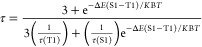
1

**Figure 9:**
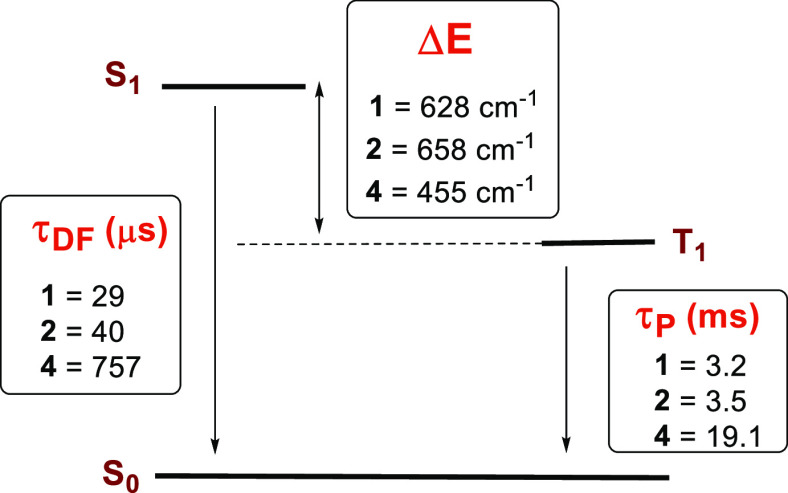
Energy level diagram for **1**, **2**, and **4** including τ_DF_ (thermally activated delayed fluorescence lifetime), τ_P_ (phosphorescence lifetime), and Δ*E*(S_1_–T_1_) calculated using eq 1. τ(S_1_) = 3.9 μs (**1**), 3.8 μs (**2**), 38.7 μs (**4**).

Comparison of the corresponding values is shown in Figure 9. Δ*E*(S_1_–T_1_) gaps for **1**, **2**, and **4** are lower than those found for the neutral compound containing a *nido*-carborane diphosphane [Cu(dppnc)(Htbz)] (925 cm^–1^ in the solid state and 1025 cm^–1^ in the PMMA film at 5 wt %), being that for **4** the smallest energy gap.

**Figure 10:**
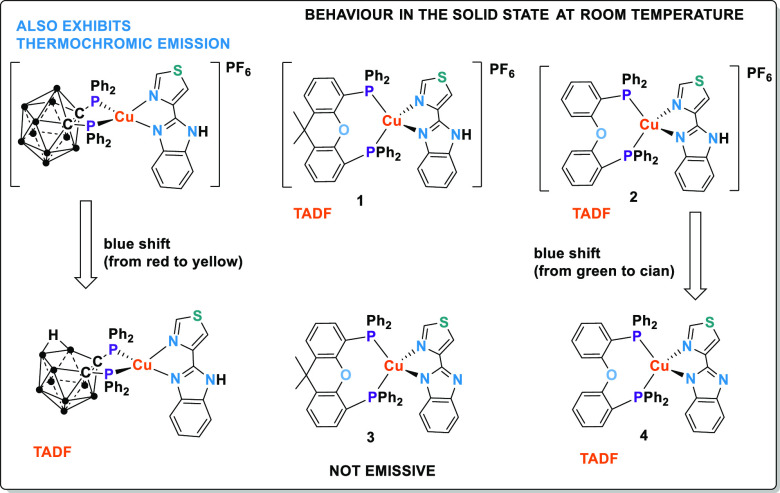
Resume of the emissive properties of complexes with the Htbz or tbz^–^ ligands and different diphosphanes.

Emission properties in the solid state of **1**–**4**, [Cu(dppcc)(Htbz)]PF_6_ and [Cu(dppnc)(Htbz)], are resumed in Figure 10. The aim is to analyze the influence of the diphosphane and neutral or cationic nature of the complexes on their emissive properties.

#### Influence of Compound Charge

The neutral nature of the compound does not guarantee TADF behavior, as the neutral [Cu(dppnc)(Htbz)] (Figure 1b) and **4** species not only display TADF but also the cationic compounds **1** and **2**.

An important difference when comparing the neutral complexes **3**, **4**, and [Cu(dppnc)(Htbz)] should be taken into account, as in compounds **3** and **4**, the negative charge is located in the diimine ligand and not in the diphosphane ligand. This leads to different frontier orbitals as shown below.

When comparing the emission energy in the solid state at room temperature of cationic complexes ([Cu(dppcc)(Htbz)]PF_6_ or **2**) with the corresponding neutral complexes ([Cu(dppnc)(Htbz)] or **4**), a blue shift is observed (Figure 10).

Quantum yields in the solid state are very low for complexes **1**, **2**, and **4** and [Cu(dppcc)(Htbz)]PF_6_ (about 1% for **1** and **2** and a bit higher, 3%, for **4**) and almost one order of magnitude increment is found for the neutral compound [Cu(dppnc)(Htbz)] (16%, powder). Hence, the quantum yield or the presence of TADF is not only ruled by the neutral or cationic nature of the complex. Furthermore, deprotonation of the Htbz ligand does not lead to an important increment of the quantum yield.

#### Influence of the Diphosphane

The emission energies and lifetimes of complexes **1** and **2** are very similar. They are blue (**1**) or green (**2**) emissive, respectively, in the range 298–77 K and display TADF. These observations suggest that the replacement of dpephos by xantphos does not have a relevant role in the luminescence of these complexes with the neutral Htbz ligand.

Changing xantphos or dpephos by the *closo*-carborane diphosphane (dppcc) leads to very different behavior, as compound [Cu(dppcc)(Htbz)]PF_6_ does not display TADF and exhibits an important thermochromism (red emissive at room temperature and yellow emissive at 77 K). Thus, among analogous cationic complexes, the one with the *closo*-carborane diphosphane exhibits very different properties.

From these data, it seems that the only factor which enhances the quantum yield is the presence of the *nido*-carborane diphosphane. In order to check if the presence of the *nido*-carborane leads to the highest quantum yield for any diimine, quantum yields of complexes with dpephos, xantphos, dppnc, and different diimines: Htbz, 2,9-dimethyl-1,10-phenantroline, or 6,6′-dimethyl-2,2′-bipyridine has been revised (see ESI) and this trend has not been found for 6,6′-dimethyl-2,2′-bipyridine.^24^

**Figure 11:**
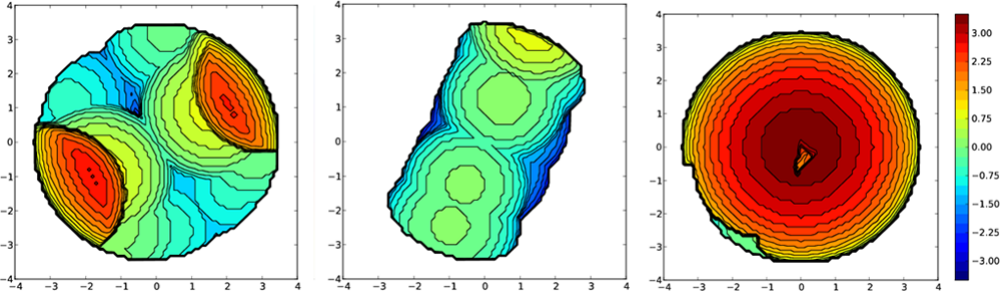
Topographical steric maps for xantphos, Htbz, and {(xantphos) + (Htbz)} for compound **1** ([Cu(P^P)(Htbz)]PF_6_).

As indicated in the Introduction section, not many studies deal with the role of the diphosphane in the emissive properties of these heteroleptic copper(I) complexes. To afford this analysis, the intuitive observation of the bulky or rigid nature of the ligand (which would avoid Jahn Teller distortion in the excited state, leading to no radiative pathways) is not a reliable guide. It has been reported than %*V*_bur_ (which represents the occupied volume by a given ligand or group of ligands inside a sphere of a defined radius around the metal center) could be an effective tool to understand which pair of diphosphane–diimine could lead to better quantum yields.^6,13^ Thus, we have calculated the %*V*_bur_ for the diphosphane, diimine, and the {(P^P) + (N^N)} unit in complexes **1**–**4**. Table 2 resumes the calculated values and Figure 11 shows, as an example, the topographical maps corresponding to the analysis of compound **1**.

**Table 2:** %*V*_bur_ for P^P, N^N, and {(P^P) + (N^N)} Units in Complexes **1**–**4** and [Cu(dppnc)(Htbz)]^+^^a^

compound	P^P^c^	N^N^c^	{(P^P) + {N^N)}^c^	Φ^c^ (%)
1^b^	58.2	32.5	89.2	1
2^b^	57.7	32.5	88.5	1
3^b^	58.5	33.0	90.0	
4^b^	57.8	33.2	89.1	3
[Cu(dppnc)(Htbz)]^c^	49.9	31.3	81.0	10

a See ESI for additional comments.

b Calculated from the crystal structure data.

c Calculated from the optimized structure.

Table 2 shows higher (P^P) %*V*_bur_ values found for xantphos than for dpephos when comparing analogous complexes. This trend is opposite to that observed for compounds with substituted 2,2′-bipyridine.^24^ High values of %*V*_bur_ for the {(P^P) + (N^N)} unit seem to lead to high quantum yields if this value is not achieved due to very high values of %*V*_bur_ for any of the bidentate ligands:(P^P) or (N^N), with 60% as the reference top value.^13^ Similar %*V*_bur_ values for the diphosphane ligands have been found in complexes **1**–**4**. The highest %*V*_bur_ value for the {(P^P) + (N^N)} and not too high %*V*_bur_ values for the [N^N] and [P^P] individual ligands have been found for compound **3**. These results would not explain the lack of emission for **3** at room temperature due to steric factors. Regarding compound [Cu(dppnc)(Htbz)], the calculated %*V*_bur_ values do not explain its higher quantum yield.

Void spaces in the solid have also been reported as relevant as allow oxygen to get into the solid which would favor emission quenching.^13,25^ The void volume for cell has been calculated using the SQUEEZE option of PLATON as 86 Å^3^ (**1**), 188 Å^3^ (**2**), 29 Å^3^ (**3**), and 16 Å^3^ (**4**), which represent 2% (**1**), 4.4% (**2**), 0.7% (**3**), and 0.8% (**4**) of the cell volume. These data do not explain the lack of emission found for **3**. Thus, a possible origin of the lack of emission at room temperature for **3** could be related with a low T_1_ energy^13^ which would favor emission quenching.

#### Theoretical Studies

In order to get insight into the differences in the frontier orbitals which could explain the observed facts, we have carried out TD-DTF theoretical studies. Comparison of experimental and optimized bond distances and angles is shown in Table 3. Optimized Cu–P and Cu–N distances are longer than the experimental ones, but the variation of α and N–Cu–N and P–Au–P bond angles is erratic.

**Table 3:** Selection of Bond Distances (Å) and Angles (°) for Complexes **1**, **2**, and **4**

compound	Cu–P	Cu–N	N–Cu–N	P–Cu–P	α^a^
**1**					
X-ray data	2.2588	2.127	80.531	116.98	82.86
	2.2374	2.076			
optimized	2.3796	2.1449	77.014	119.868	89.62
	2.3687	2.3068			
**2**					
X-ray data	2.244	2.153	79.2	116.76	86.93
	2.271	2.059			
optimized	2.3841	2.2956	76.88	116.508	82.91
	2.3659	2.1451			
**4**					
X-ray data	2.2289	2.1372	80.63	115.068	89.23
	2.2580	2.0026			
optimized	2.2467	2.3834	80.34	113.699	87.54
	2.0553	2.3846			

a α = Angle between N–Cu–N and P–Cu–P planes.

**Figure 12:**
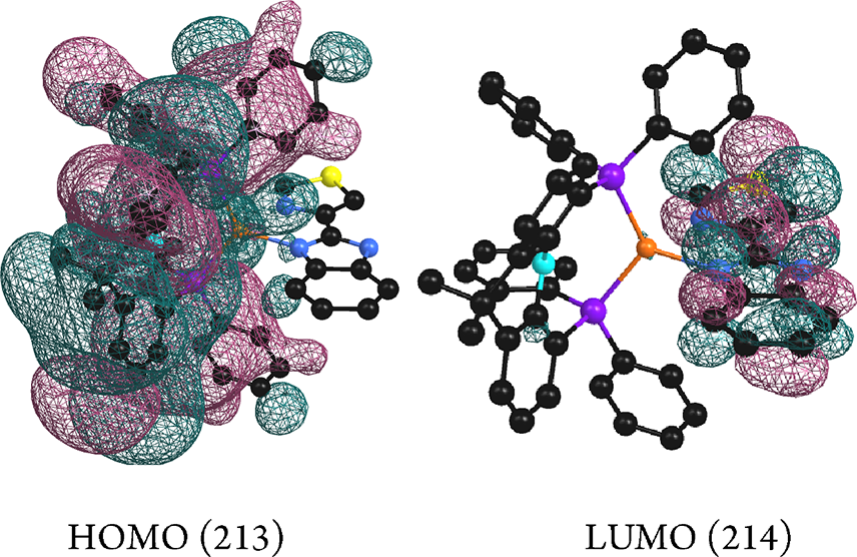
HOMO and LUMO calculated molecular orbitals for compound **1**.

For compound **1**, the S_0_ → S_1_ transition corresponds to that of the HOMO → LUMO orbitals at the ground state (Table 4, Figure 12). The electronic density of the HOMO is mostly located in the copper atom (6%) and the diphosphane. The major contribution corresponds to the diphosphane skeleton (42%), that of the phosphorus atoms (7%), and that of the phenyl rings at phosphorus (1%). The electronic density of the LUMO is mostly located in the skeleton of the diimine (87%) with contribution of the nitrogen atoms of 7%. Consequently, the nature of the transition which originates the absorption leading to the emission may be attributed to a mostly ligand L (diphosphane) to ligand (L′) diimine, charge transfer transition (LL′CT), although the copper atom slightly contributes to the HOMO orbital, leading to a MLL′CT nature.

**Table 4:** Calculated Transitions for **1**, **2**, and **4**

	λ_ex_^a^	transition (f)		orbitals	λ^b^
**1**	400^c^	S_0_ → S_1_	0.0088	HOMO (213) → LUMO (214)	412
**2**	400	S_0_ → S_1_	0.0078	HOMO (202) → LUMO (203)	416
**4**	365	S_0_ → S_1_	0.0016	HOMO (202) → LUMO (203)	409
		S_0_ → S_8_	0.0031	HOMO (202) → LUMO+1 (204)	367
				HOMO (202) → LUMO+3 (206)	
				HOMO (202) → LUMO+4 (207)	

a λ_ex_ = excitation maximum in the solid state (nm).

b λ = calculated energy (nm).

c Broad band from 332 to 400 nm.

**Figure 13:**
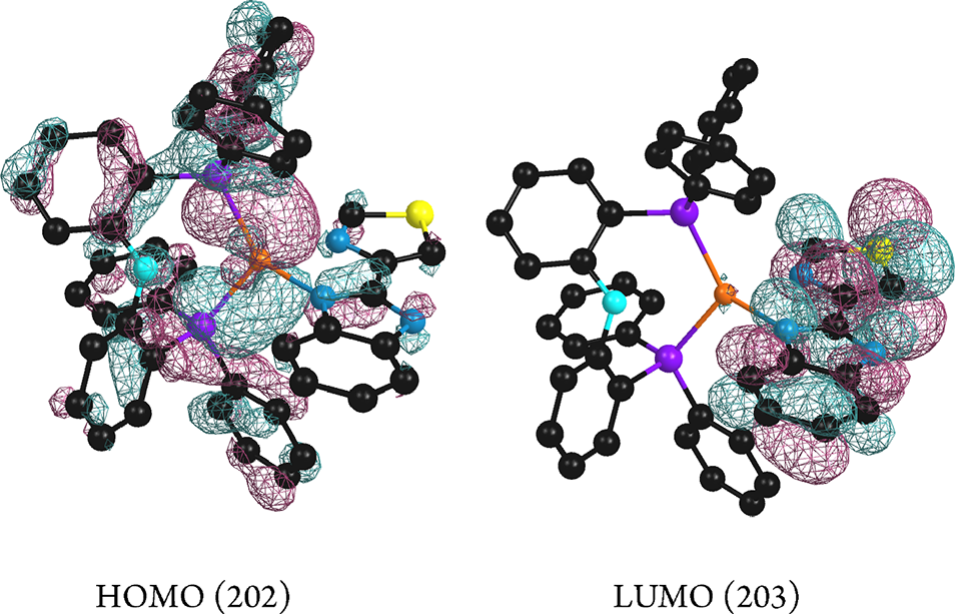
HOMO and LUMO calculated molecular orbitals for compound **2**.

For compound **2**, transition S_0_ → S_1_ corresponds to HOMO–LUMO transition at the ground state (Table 4, Figure 13). The copper atom and diphosphane contribute mostly to the HOMO orbital (37 and 54%, respectively). Contribution to the LUMO electronic density is mainly located in the Htbz ligand (97%). Thus, a metal–ligand (L, diphosphane) to ligand (L′, diimine) charge transfer (MLL′CT) is responsible for the excitation which originates the emission in **2** with more important contribution of copper than in **1**.

**Figure 14:**
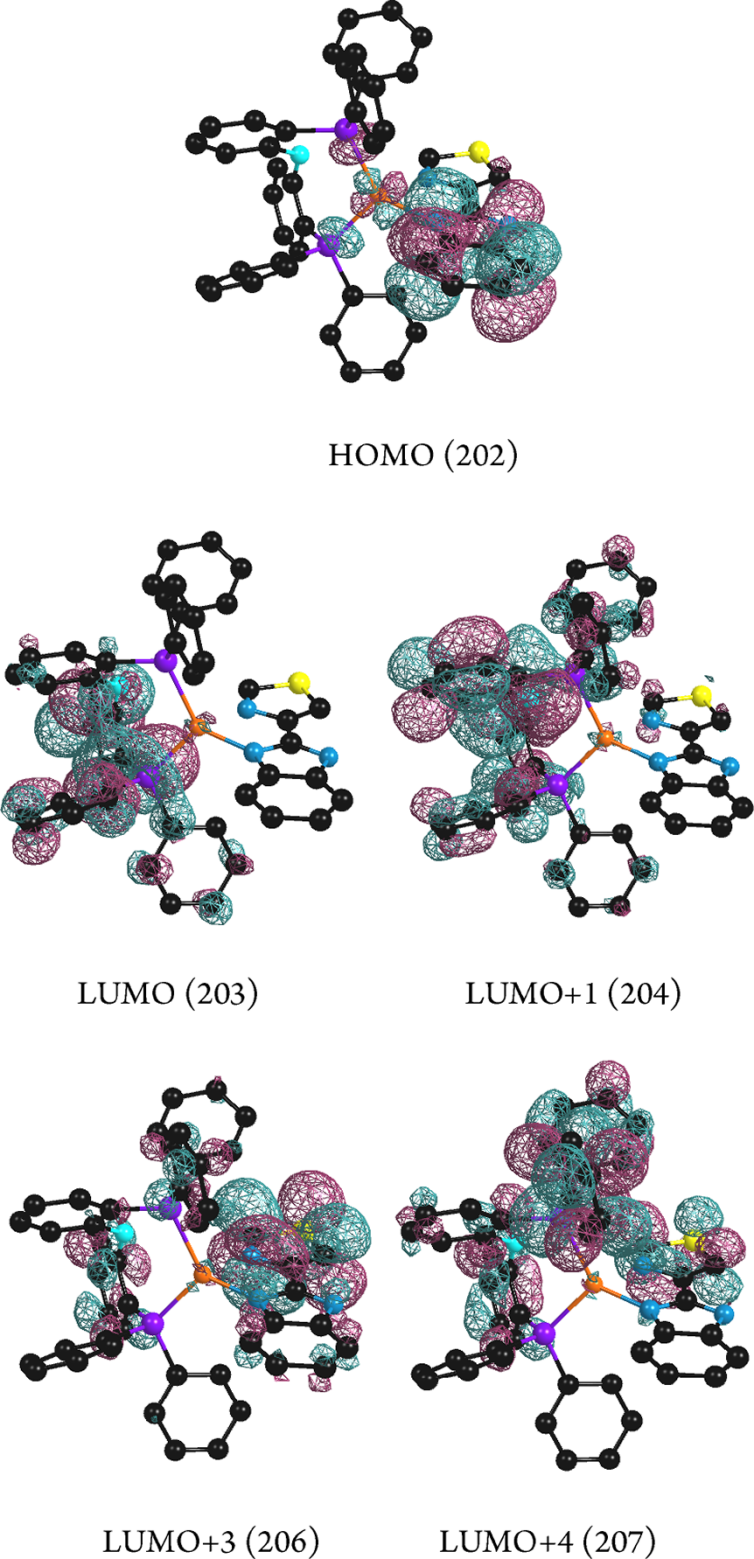
HOMO, LUMO, and LUMO+*n* calculated molecular orbitals for compound **4**.

For compound **4**, the electronic density corresponding to the HOMO orbital is mostly located in the (tbz^–^) ligand (76%) and the copper atom (13%). That of the LUMO is located mainly in the phosphane (36%). Transition S_0_ → S_1_ corresponds to HOMO–LUMO transition at the ground state. Excitation maximum energy better fits with a transition which involves the HOMO and LUMO+1, LUMO+3, and LUMO+4 with more participation of the LUMO+4 orbital (Tables 4 and 5, Figure 14). LUMO+1 electronic density is mainly located in the diphosphane ligand, but in LUMO+3 and LUMO+4, the electronic density is located both in the diphosphane and diimine ligands. Thus, for compound **4** the transition responsible of the absorption leading to the emission may be attributed to a mixture of a metal–ligand (L) (diimine) to ligand (L′) (diphosphane) charge transfer transition (MLL′CT) and intraligand IL(diimine) transition.

**Table 5:** Contributions of the Different Parts of the Molecule to the Frontier Orbitals in **4**

	percentage (%)		
orbital	Cu	diimine	diphosphane
LUMO+1 (204)	3	4	67
LUMO+3 (206)	1	55	13
LUMO+4 (207)		13	61

Metal–ligand (diphosphane) to ligand (diimine) transitions have been extensively found as responsible of the origin of the absorption leading to luminescence in tetracoordinate heteroleptic diimine-diphosphane copper complexes.^4,6,14,15^ Such origin has been found for complexes [Cu(dppnc)(Htbz)] and [Cu(dppcc)(Htbz)]PF_6_ with an important reduction of the copper participation in the neutral complex, which exhibits TADF.^11^ In this case, we are particularly interested in the effect of ligand Htbz deprotonation.

Comparison of complexes **2** and **4** shows that orbital composition is very different, metal participation in the frontier orbitals deeply diminish upon deprotonation in **4**, and the transition may be better defined as mixed ML (diimine) to L′ (diphosphane) charge transfer transition and intraligand (diimine) transition, instead of the ML (diphosphane) to L′ (diimine) nature found in **2**. These changes could be related with the reduction of the Δ*E*(S_1_–T_1_) gap and the blue shift of the emission maximum found for **4**, compared to that observed for **2**.

## Experimental Section

### Instrumentation

NMR spectra were carried out in a Bruker AV 400 or 300 in CDCl_3_ if the solvent is not specified and chemical shifts (ppm) reported relative to the solvent peaks of the deuterated solvent.^26^ Thermogravimetric analyses were carried out in a TA Instruments SDT2960 equipment at a rate of 10 °C min^–1^ under a nitrogen atmosphere until 600 °C and under an air atmosphere from 600 to 750 °C.

Steady-state photoluminescence spectra were recorded with a Jobin-Yvon Horiba Fluorolog FL-3-11. Lifetime measurements were recorded with a Fluoromax phosphorimeter accessory containing a UV xenon flash tube or a Horiba Jobin Ybon LED with a pulse duration <1.2 ns. Frequencies of the LED used were selected attending to the excitation energies. An OptistatDN Oxford variable temperature liquid nitrogen cryostat has been used for lifetime studies at different temperatures and a liquid nitrogen dewar assembly for steady-state studies at 77 K. Quantum yields were measured by the absolute method using a Hamamatsu Quantaurus-QY C11347 compact one-box absolute quantum yield measurement system. In order to prove the reproducibility of the measurements, three or more measurements were carried out for each compound. Through studies carried out for different substances using both, the absolute method and the comparative one, the relative uncertainty for the absolute method has been determined as less than 6%.^27^

### Crystallography

Crystals suitable for X ray studies were obtained by diffusion of *n*-hexane over a solution of the corresponding compound in dichloromethane or acetone. Crystals were mounted in inert oil on a glass fiber and transferred to the cold gas stream of a SMART APEX (**1**, **3**, and **4**) or mounted on a MiTeGen Crystal micromount and transferred to the cold gas stream of a Bruker D8 VENTURE (**2**) diffractometer. Data were collected using monochromated MoKα radiation (λ = 0.71073 Å). Scan-type ω. Absorption correction based on multiple scans was applied with the program SADABS.^28^ The structures were refined on F2 using the program SHELXL-2018.^29^ All non-hydrogen atoms were refined anisotropically. Hydrogen atoms were included using a riding model. CCDC depositions 2220908 (**4**), 2220909 (**1**), 2220910 (**2**), and 2220911 (**3**) contain the supplementary crystallographic data. These data can be obtained free of charge by The Cambridge Crystallography Data Center.

### Theoretical Studies

The Gaussian 09 program was used to carry out the TD-DTF calculations. For comparison purposes, the studies have been carried out using the same functional and basis set than those reported for complexes [Cu(dppcc)(Htbz)][PF_6_] and [Cu(dppnc)(Htbz)]^+^.^17^ Geometry optimizations were performed on the ground state using the hybrid B3LYP functional. The basis set def2-SVP (for C, S, and H atoms),^7^ def2-TZVP (for P, N, and O atoms), and LANL2DZ for the copper atom.^12,13^ For the copper atom, the corresponding associated pseudopotential to LANL2DZ was applied.

### Calculation of the Buried Volume

Calculations of the Buried volume^30^ and topographical steric maps^31^ were calculated and created, respectively, using the SambVca 2.1 package^32^ (https://www.molnac.unisa.it/OMtools/sambvca2.1/index.html). The center of the sphere was defined by the copper atom. For the calculation of the %*V*_bur_(P^P), the P atoms of the diphosphane were selected to define the negative *Z*-axis and the N atoms coordinated to the copper center of the diimine ligand to define the *XZ*-plane. All atoms except those of the diphosphane ligand were deleted. For the calculation of the %*V*_bur_(N^N), the N atoms of the diimine coordinated to the copper center were used to define the negative Z-axis and the P atoms of the diphosphane to define the *XZ*-plane. All the atoms except those of the diimine ligand were deleted. For the calculation of the %*V*_bur_{(P^P) + (N^N)}, the N atoms of the diimine coordinated to the copper center were used to define the negative *Z*-axis and the P atoms of the diphosphane to define the *XZ*-plane. The copper atom was deleted. For all the calculations, the selected parameters were Bondi radii 1.17; sphere radius *r* = 3.5 Å; Mesh spacing 0.10, and the H atoms excluded.

### Synthesis of Complexes [Cu(P^P)(N^N)]

#### General Procedures

The synthetic procedures were carried out under an Ar atmosphere, using Schlenk techniques. Dry degassed solvents were used. The starting materials [Cu(CH_3_CN)_4_]PF_6_, Htbz, xantphos, and dpephos are commercially available and were used as received.

##### Synthesis of [Cu(P^P)(Htbz)]PF_6_ [P^P = Xantphos (1), Dpephos (2)]

Complexes were synthesized following reported procedures. To a dichloromethane (ca. 20 mL) solution of [Cu(CH_3_CN)_4_]PF_6_ (0.1 mmol, 37.2 mg), the corresponding diphosphane was added [0.1 mmol: P^P = xantphos, 57.8 mg; P^P = dpephos, 53.7 mg]. The mixture was stirred for thirty minutes and Htbz (0.1 mmol, 20.1 mg) was added. After one hour, the solution was evaporated to minimum volume (ca. 2 mL). Addition of *n*-hexane (ca. 20 mL) led to the precipitation of a white-yellow pale solid, corresponding to **1** or **2**, which was filtered and dried under vacuum.

(**1**) Yield: 81.9 mg, 83%. ^1^H NMR (400 MHz, {CO(CH_3_)_2_}-d6, 25 °C): δ (ppm) = 12.89 (s, 1H, HN-Htbz), 9.31 (s, 1H, Htbz), 8.58 (s, 1H, Htbz), 7.87 (dd, *J* = 7.8, 1.2 Hz, 2H), 7.64 (d, *J* = 8.1 Hz, 1H), 7.41–7.20 (m, 15H), 7.16 (m, 4H), 7.00–6.86 (m, 5H), 6.68–6.57 (m, 2H), 6.44 (m, 1H), 1.98 (s, 3H, CH_3_-xantphos), 1.67 (s, 3H, CH_3_-xantphos). ^31^P{^1^H} NMR (162 MHz, {CO(CH_3_)_2_}-d6, 25 °C): δ (ppm) = −13.3. Q-TOF *m*/*z*: [M-PF_6_]^+^_Experimental_ 842.1547, [M-PF_6_]^+^_Calculated_ 842.1795.

(**2**) Yield: 75.1 mg, 79%. ^1^H NMR (400 MHz, {CO(CH_3_)_2_}-d6, 25 °C): δ (ppm) = 12.88 (s, 1H, HN-Htbz), 9.29 (s, 1H, Htbz); 8.53 (s, 1H, Htbz), 7.66 (d, *J* = 7.9 Hz, 1H), 7.45–7.05 (m, 29H), 6.93–6.74 (m, 2H). ^31^P{^1^H} NMR (162 MHz, {CO(CH_3_)_2_}-d6, 25 °C): δ (ppm) = −13.1. Q-TOF *m*/*z*: [M-PF_6_]^+^_Experimental_ 802.1290, [M-PF_6_]^+^_Calculated_ 802.1266.

##### Synthesis of [Cu(P^P)(tbz)] [P^P = Xantphos (**3**), Dpephos (**4**)]

To a suspension of 0.1 mmol of **1** (99.5 mg) or **2** (95.5 mg) in methanol (ca. 20 mL), KOH (1.5 mmol, 84.2 mg) was added. The mixture was refluxed for 5 h. The resulting solid was filtered, washed with methanol (3 × 5 mL), and dried under vacuum.

(**3**) Yield: 46.3 mg, 61%. ^1^H NMR (400 MHz, {CH_2_Cl_2_}-d2, 25 °C): δ (ppm) = 8.03 (s, 2H), 7.63 (dd, *J* = 7.8, 1.3 Hz, 2H), 7.55 (d, *J* = 8.0 Hz, 1H), 7.28–7.14 (m, 4H), 7.14–6.95 (m, 18H), 6.87 (t, *J* = 7.6 Hz, 1H), 6.63 (t, *J* = 7.6 Hz, 1H), 6.51 (d, *J* = 7.5 Hz, 1H), 6.47–6.38 (m, 2H), 1.82 (s, 3H, CH_3_-xantphos), 1.74 (s, 3H, CH_3_-xantphos). ^31^P{^1^H} NMR (162 MHz, {CH_2_Cl_2_}-d2, 25 °C): δ (ppm) = −14.3. Q-TOF *m*/*z*: [M + H]^+^_Experimental_ 842.1542, [M + H]^+^_Calculated__842.1579.

(**4**) Yield: 33.1 mg, 49%. ^1^H NMR (400 MHz, {CO(CH_3_)_2_}-d6, 25 °C): δ (ppm) = 8.74 (s, 1H-tbz^–^), 7.95 (s, 1H, tbz^–^), 7.51 (s, 1H), 7.36 (m, 2H), 7.33–7.09 (m, 23H), 7.00 (t, *J* = 7.2 Hz, 2H), 6.82 (s, 1H), 6.78–6.65 (m, 3H). ^31^P{^1^H} NMR (162 MHz, {CO(CH_3_)_2_}-d6, 25 °C): δ (ppm) = −14.9. Q-TOF *m*/*z*: [M + H]^+^_Experimental_ 802.1242, [M + H]^+^_Calculated_ 802.1266.

## Conclusions

Influence of the diphosphane and deprotonation of the Htbz ligand in the emissive behavior of [Cu(P^P)(Htbz)]PF_6_ complexes has been studied.

Among the cationic complexes, substitution of the carborane diphosphane dppcc (studied in a previous work) by dpephos and xantphos in [Cu(P^P)(Htbz)]PF_6_ does not lead to an increment in the quantum yield. Complexes with dpephos and xantphos exhibit TADF while the compound with dppcc exhibits strong thermochromism. For these cationic [Cu(P^P)(Htbz)]PF_6_ compounds, TD-DFT calculations indicate that substitution of xantphos by dpephos leads to different participation of the copper atom in the transitions responsible of the absorption, leading to luminescence. For both complexes, such transition may be considered as originated by a metal–ligand (L, diphosphane) to ligand (L′, diimine) charge transfer transition (MLL′CT): with a very small contribution of the copper atom in the HOMO orbital (6%) when P^P = xantphos and a more important contribution of the copper atom if P^P = dpephos (37%). Higher contribution of the metal atom has not led to higher quantum yield, and both complexes display TADF.

Comparison of the TD-DFT calculations of [Cu(dpephos)(Htbz)]PF_6_ and [Cu(dpephos)(tbz)] shows that deprotonation of the diphosphane leads to important changes in the origin of the transitions responsible of the absorption, leading to luminescence. A metal–ligand (L, diimine) to L′ (diphosphane) charge transfer (MLL′CT) mixed with an intraligand IL (diimine) origin may be proposed for the absorption transition which originates the emission in the neutral complex. Thus, deprotonation leads to the substitution of the diphosphane by the diimine in the HOMO departure orbital and a relevant role of the diphosphane, retaining the diimine presence, in the arrival orbitals. TADF is observed for both complexes with a diminishment of the Δ*E*(S_1_–T_1_) gap upon deprotonation.

## Supporting Information Available

Additional details of the thermogravimetric analysis, emissive properties, topographical steric maps, and theoretical studies (PDF)
Coordinates of the optimized structures (XYZ)

## Supplementary Material

ic3c01409_si_001.pdf

ic3c01409_si_002.xyz
