# Supplementary material for: Thermally Activated Delayed Fluorescence in Neutral and Cationic Copper(I) Complexes with the 2-(4-Thiazolyl)benzimidazole Ligand
Source: Inorg Chem. 2023 Jun 22;62(26):10431–9. doi: 10.1021/acs.inorgchem.3c01409 (PMC10324396; doi:10.1021/acs.inorgchem.3c01409)
Supplement: Supplementary file 1 — ic3c01409_si_001.pdf [file ic3c01409_si_001.pdf]

ESI

THERMALLY ACTIVATED DELAYED FLUORESCENCE (TADF) IN NEUTRAL AND CATHIONIC COPPER(I) COMPLEXES WITH THE 2-(4-THIAZOLYL)BENZIMIDAZOLE LIGAND

Adrián Alconchel, Olga Crespo,* M. Concepción Gimeno*

Departamento de Química Inorgánica, Instituto de Síntesis Química y Catálisis Homogénea (ISQCH). Universidad de Zaragoza-CSIC. E-50009 Zaragoza, Spain.

*Email: ocrespo@unizar.es (O. C.), gimeno@unizar.es (M. C. G)

Figure S1. TGA curve of **1**.

Figure S2. TGA curve of **2**.

Figure S3. TGA curve of **3**.

Figure S4. TGA curve of **4**.

Figure S5. Emission and excitation spectra of **1** in solid state at room temperature. Emission spectrum recorded at excitation λ of 360 nm, excitation spectrum recorded at emission λ of 515 nm.

Figure S6. Emission and excitation spectra of **1** in solid state at 77 K. Emission spectrum Emission spectrum recorded at excitation λ of 360 nm, excitation spectrum recorded at emission λ of 505 nm.

Figure S7. Emission and excitation spectra of **2** in solid state at room temperature. Emission spectrum recorded at excitation λ of 390 nm, Emission spectrum recorded at excitation λ of 570 nm.

Figure S8. Emission and excitation spectra of **2** in solid state at 77 K. Emission spectrum recorded at excitation λ of 390 nm, excitation spectrum recorded at emission λ of 510 nm.

Figure S9. Emission and excitation spectra of **3** in solid state at 77 K. Emission spectrum recorded at excitation λ of 330 nm, excitation spectrum recorded at emission λ of 484 nm.

Figure S10. Emission and excitation spectra of **4** in solid state at room temperature. Emission spectrum recorded at excitation λ of 360 nm, excitation spectrum recorded at emission λ of 495 nm.

Figure S11. Emission and excitation spectra of **4** in solid state at 77 K. Emission spectrum recorded at excitation λ of 360 nm, excitation spectrum recorded at excitation λ of 550 nm.

Figure S12. Decay curve and fitting data for complex **1** in solid state at room temperature (λex = 360 nm; λem = 505 nm).

Figure S13. Decay curve and fitting data for complex **1** in solid state at 77 K (λex 360 = nm; λem = 500 nm).

Figure S14. Decay curve and fitting data for complex **2** in solid state at room temperature (λex = 400 nm; λem = 520 nm).

Figure S15. Decay curve and fitting data for complex **2** in solid state at 77 K (λex = 385 nm; λem = 510 nm).

Figure S16. Decay curve and fitting data for complex **3** in solid state at 77 K (λex = 330 nm; λem = 485 nm).

Figure S17. Decay curve and fitting data for complex **4** in solid state at room temperature (λex = 360 nm; λem = 495 nm).

Figure S18. Decay curve and fitting data for complex **4** in solid state at 77 K (λex = 370 nm; λem = 492 nm).

Figure S19. Topographical steric maps for xantphos, Htbz and {(xantphos)+(Htbz)} for compound **1**.

Figure S20. Topographical steric maps for dpephos, Htbz and {(dpephos)+(Htbz)} for compound **2**.

Figure S21. Topographical steric maps for xantphos, tbz^- and {(xantphos)+(tbz^-)} for compound **3**.

Figure S22. Topographical steric maps for dpephos, tbz^- and {(dpephos)+(tbz^-)} for compound **4**.

Table S1. $\%V_{bur}$ for P^P, N^N and {(P^P)+(N^N)} units in complexes **1**-**4** and $[Cu(dppnc)(Htbz)]^+$ from optimized structures. Values are smaller than those calculated from the crystal structure data but lower values of $\%V_{bur}$ are again found for the {(P^P)+(N^N)} and (P^P) units in [Cu(dppnc)(Htbz)].

Table S2. Quantum yields [Φ (%)] for different $[Cu(P^\wedge P)(N^\wedge N)]^{0/+}$ complexes.

Table S3. Energy values for **1**, **2** and **4**.

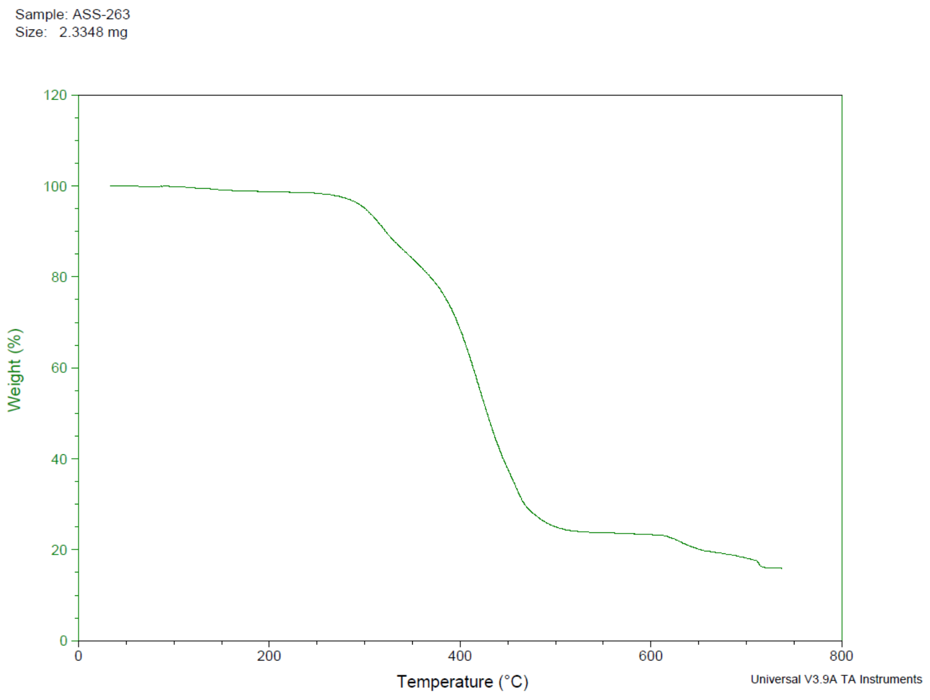

Figure S1. TGA curve of **1**

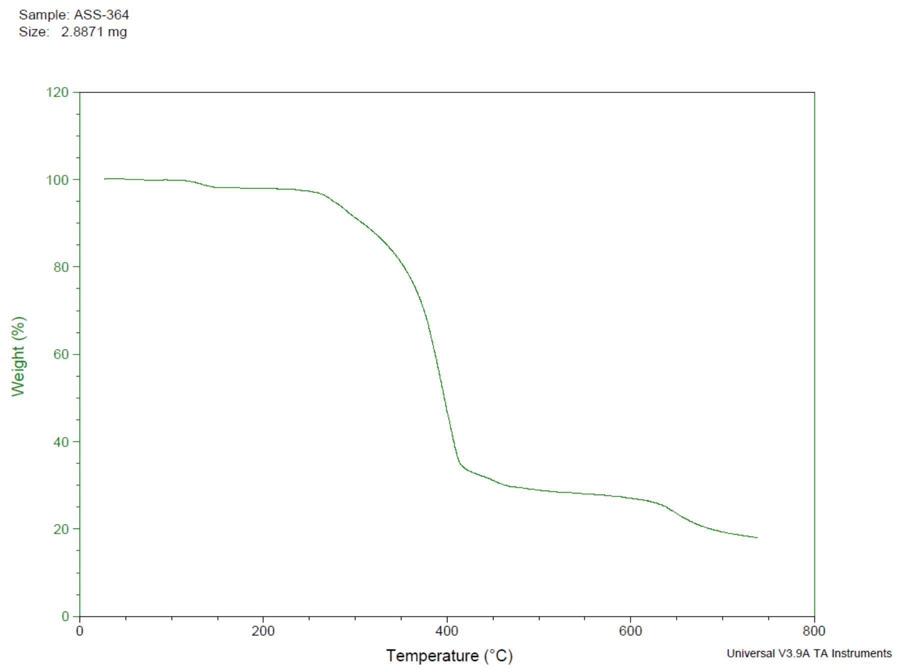

Figure S2. TGA curve of **2**

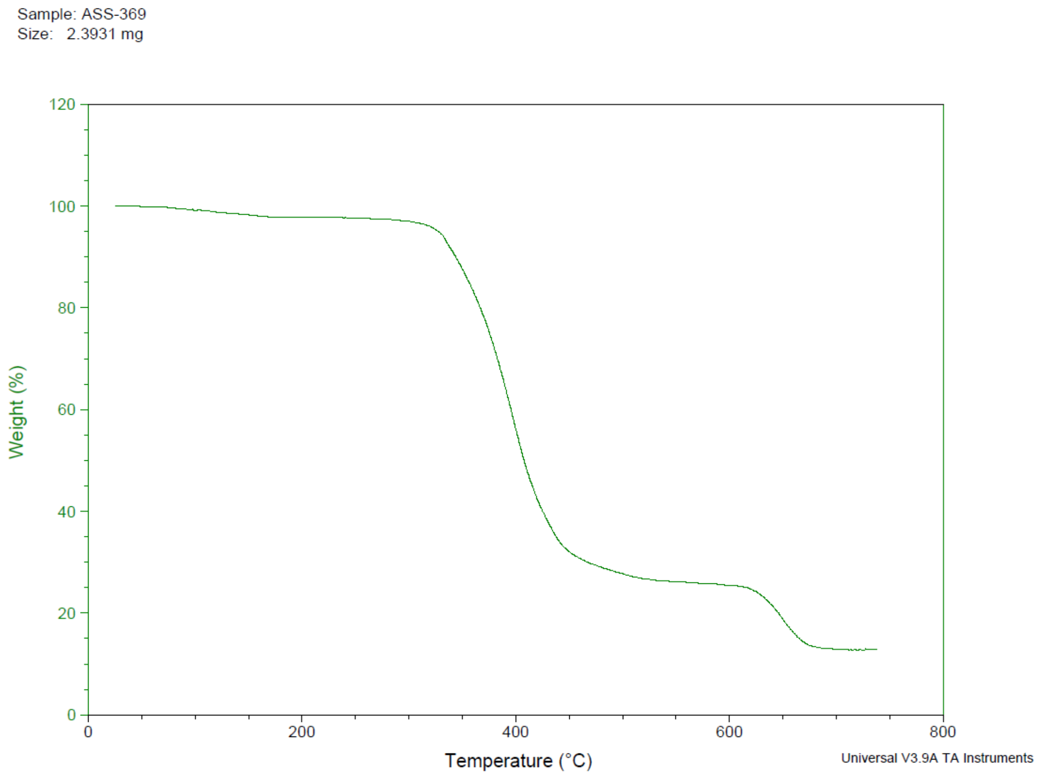

Figure S3. TGA curve of **3**

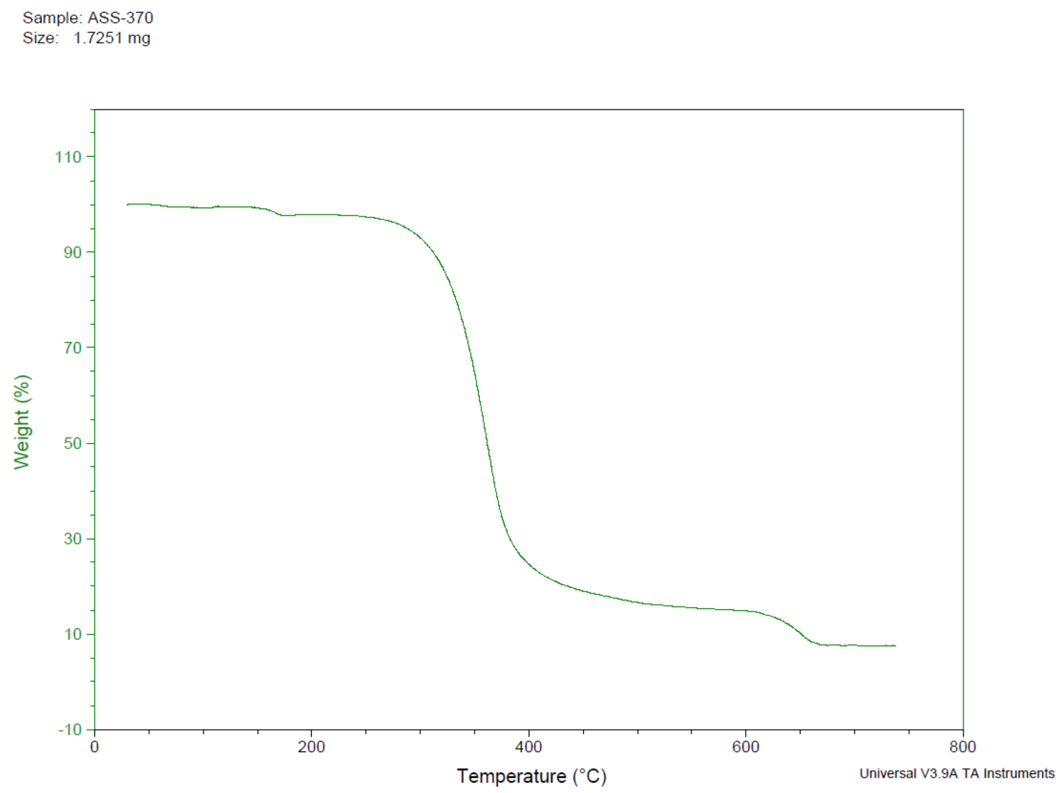

Figure S4. TGA curve of **4**

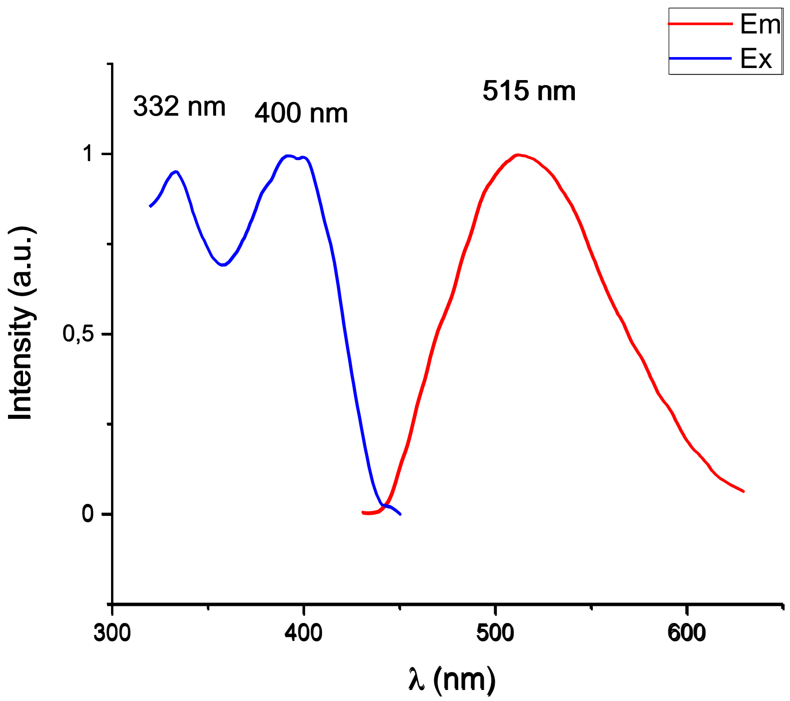

Figure S5. Emission and excitation spectra of **1** in solid state at room temperature. Emission spectrum recorded at excitation λ of 360 nm, excitation spectrum recorded at emission λ of 515 nm.

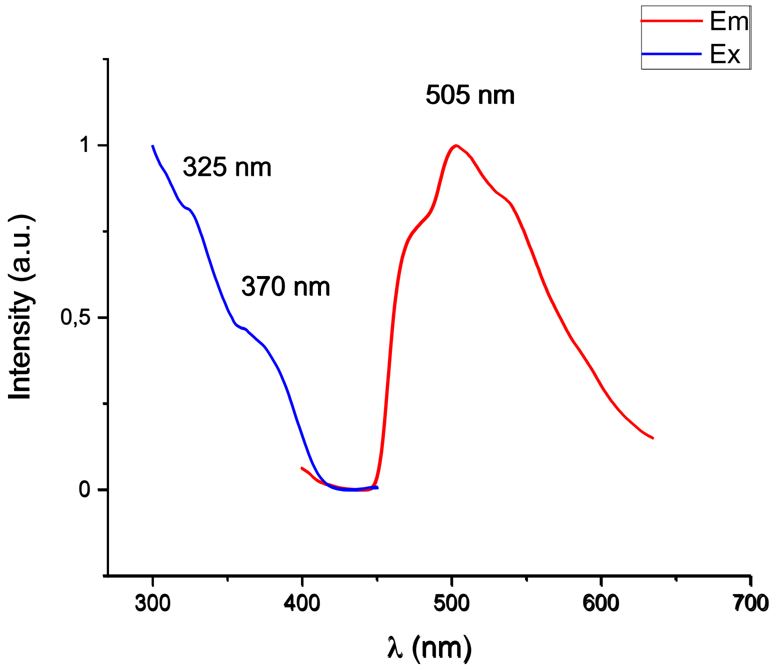

Figure S6. Emission and excitation spectra of **1** in solid state at 77 K. Emission spectrum Emission spectrum recorded at excitation λ of 360 nm, excitation spectrum recorded at emission λ of 505 nm.

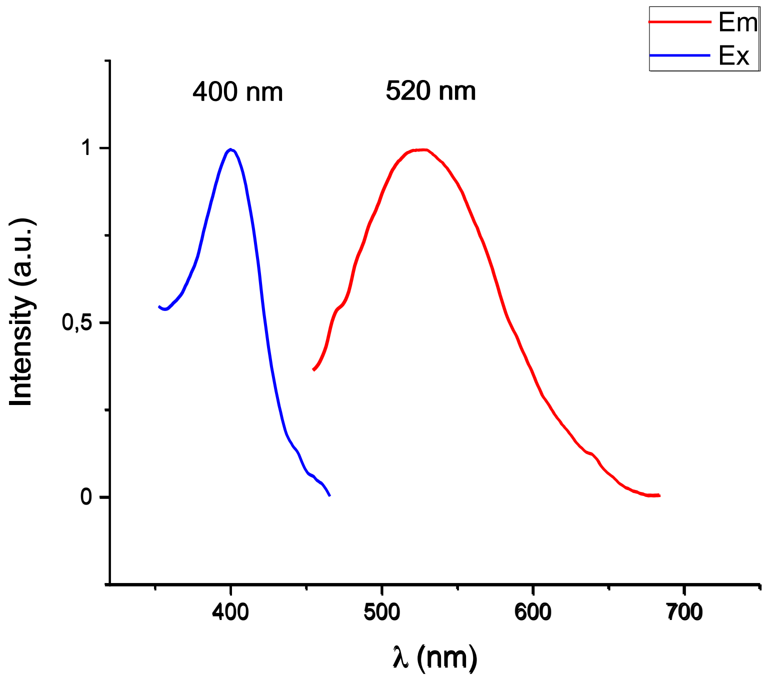

Figure S7. Emission and excitation spectra of **2** in solid state at room temperature. Emission spectrum recorded at excitation λ of 390 nm, Emission spectrum recorded at excitation λ of 570 nm.

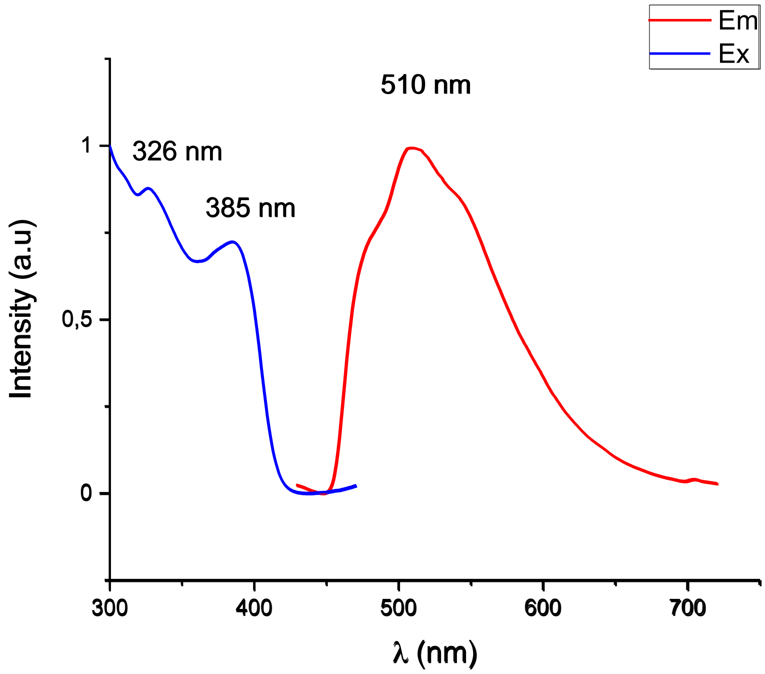

Figure S8. Emission and excitation spectra of **2** in solid state at 77 K. Emission spectrum recorded at excitation λ of 390 nm, excitation spectrum recorded at emission λ of 510 nm.

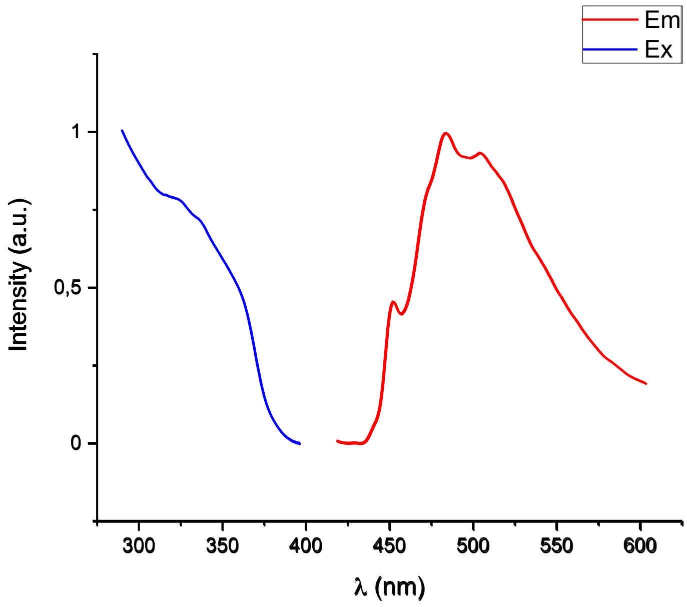

Figure S9. Emission and excitation spectra of **3** in solid state at 77 K. Emission spectrum recorded at excitation λ of 330 nm, excitation spectrum recorded at emission λ of 484 nm.

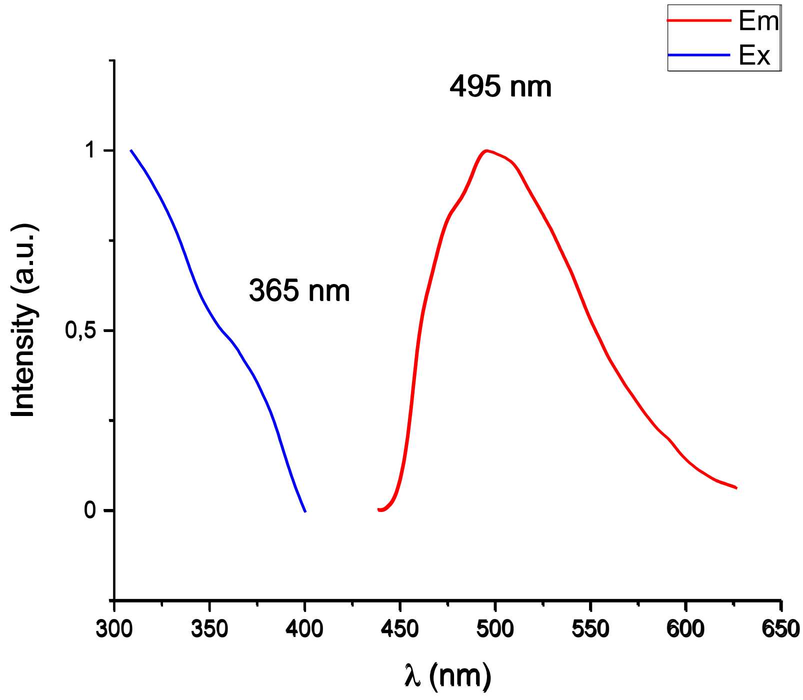

Figure S10. Emission and excitation spectra of **4** in solid state at room temperature. Emission spectrum recorded at excitation λ of 360 nm, excitation spectrum recorded at emission λ of 495 nm.

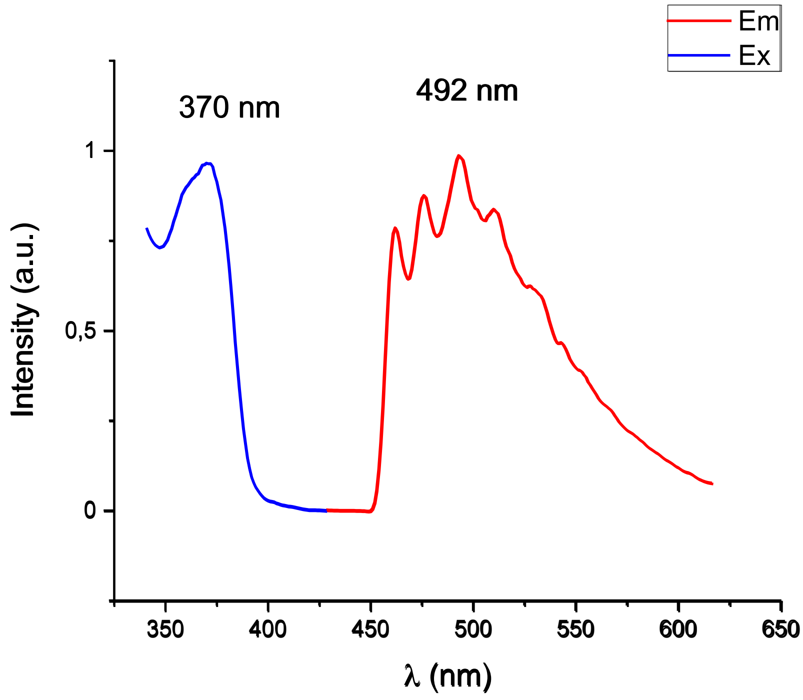

Figure S11. Emission and excitation spectra of **4** in solid state at 77 K. Emission spectrum recorded at excitation λ of 360 nm, excitation spectrum recorded at excitation λ of 550 nm.

	y0	y0	A1	A1	t1	t1	Statistics	Statistics
	Value	Standard Er	Value	Standard Er	Value	Standard Er	Reduced C	Adj. R-Squa
S1	1,95855	0,23139	3062,9610	58,3006	0,02906	2,76958E-4	7,65449	0,99713

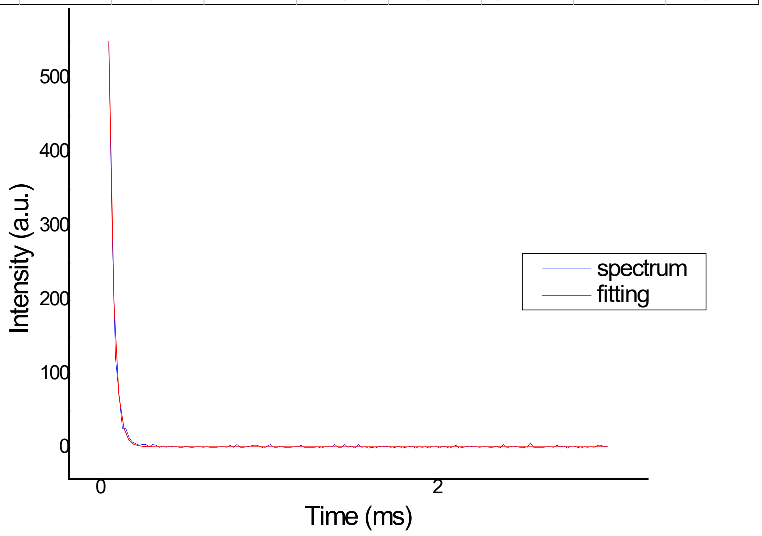

Figure S12. Decay curve and fitting data for complex **1** in solid state at room temperature (λex = 360 nm; λem = 505 nm).

	y0	y0	A1	A1	t1	t1	Statistics	Statistics
	Value	Standard Er	Value	Standard Er	Value	Standard Er	Reduced Ch	Adj. R-Squa
S1	14204,1168	164,14612	294886,757	917,79986	3,39519	0,01675	7145763,51	0,99767

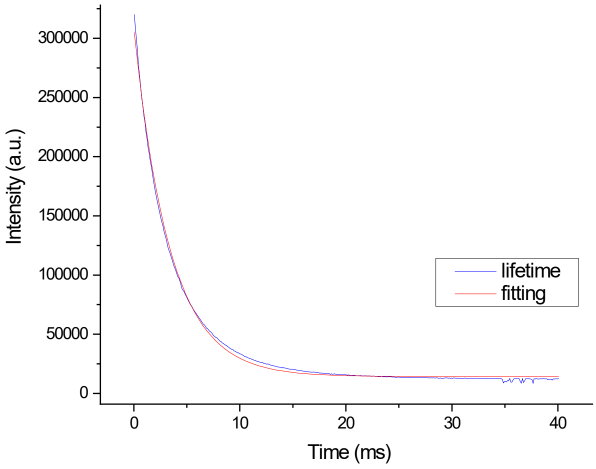

Figure S13. Decay curve and fitting data for complex **1** in solid state at 77 K (λex 360 = nm; λem = 500 nm).

	y0	y0	A1	A1	t1	t1	Statistics	Statistics
	Value	Standard Err	Value	Standard Err	Value	Standard Err	Reduced Chi	Adj. R-Squar
S1	14,09824	0,40708	2549,29431	45,73812	0,04034	4,54416E-4	24,65323	0,99546

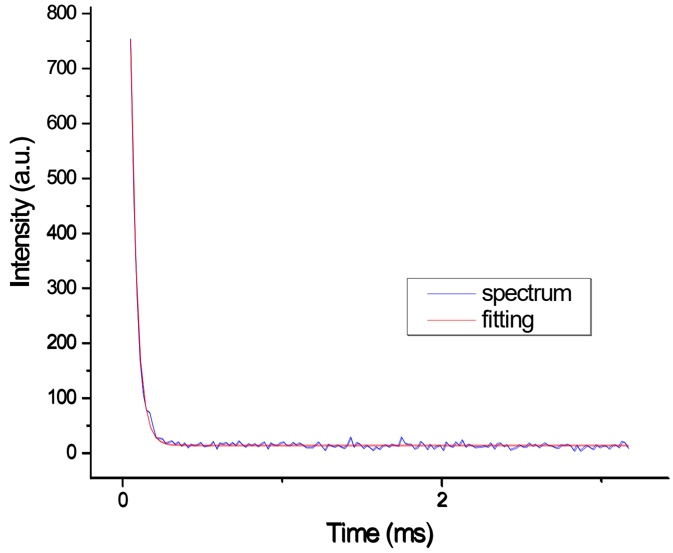

Figure S14. Decay curve and fitting data for complex **2** in solid state at room temperature (λex = 400 nm; λem = 520 nm).

	y0	y0	A1	A1	t1	t1	Statistics	Statistics
	Value	Standard E	Value	Standard E	Value	Standard E	Reduced C	Adj. R-Squ
S1	2108,2202	58,9003	133333,48	320,67242	3,5159	0,01348	903368,83	0,9986

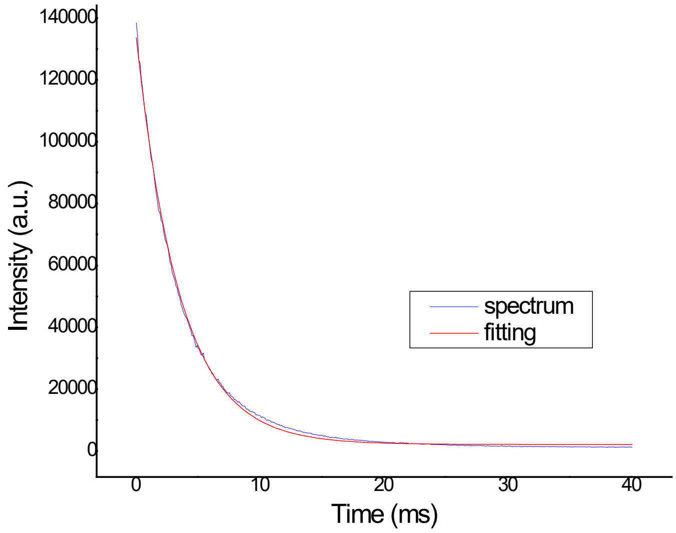

Figure S15. Decay curve and fitting data for complex **2** in solid state at 77 K (λex = 385 nm; λem = 510 nm).

	y0	y0	A1	A1	t1	t1	Statistics	Statistics
	Value	Standard Err	Value	Standard Err	Value	Standard Err	Reduced Ch	Adj. R-Squa
S1	6654.77947	128.64111	92788.3068	371.90666	20.13359	0.1572	3512129.32	0.99279

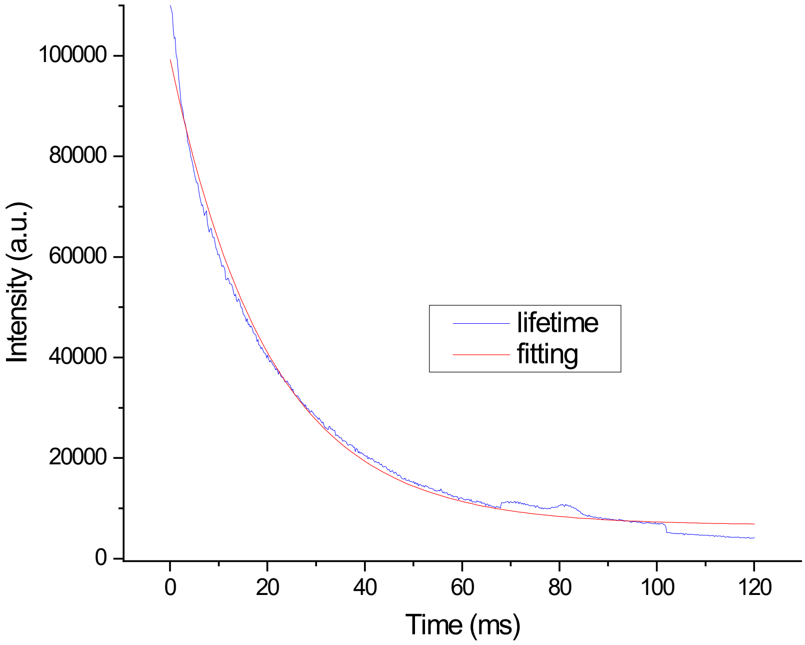

Figure S16. Decay curve and fitting data for complex **3** in solid state at 77 K (λex = 330 nm; λem = 485 nm).

	y0	y0	A1	A1	t1	t1	Statistics	Statistics
	Value	Standard Er	Value	Standard Er	Value	Standard Er	Reduced C	Adj. R-Squ
S1	528,55274	25,40911	38359,034	171,09895	0,75669	0,00499	223812,73	0,99479

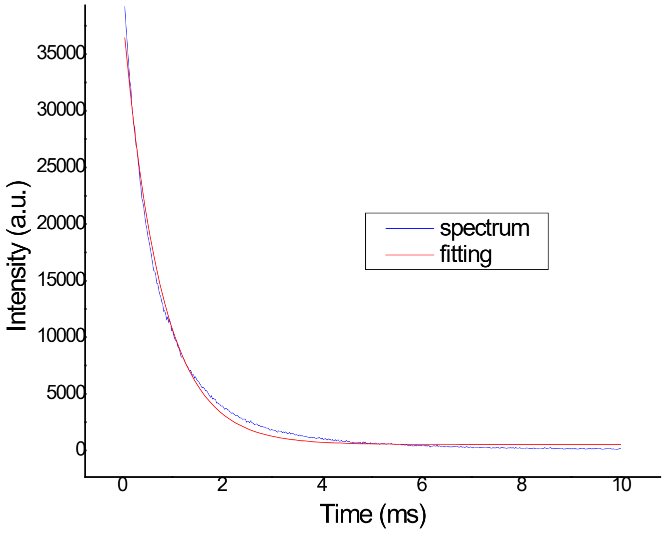

Figure S17. Decay curve and fitting data for complex **4** in solid state at room temperature (λex = 360 nm; λem = 495 nm).

	y0	y0	A1	A1	t1	t1	Statistics	Statistics
	Value	Standard Er	Value	Standard Er	Value	Standard Er	Reduced C	Adj. R-Squa
S1	-4819,0258	808,9658	370400,586	1136,71813	19,15122	0,15967	2,91907E7	0,9968

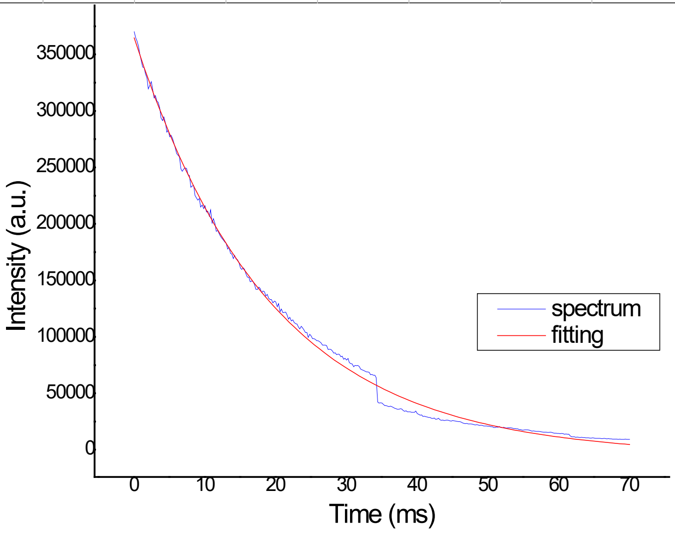

Figure S18. Decay curve and fitting data for complex **4 i**n solid state at 77 K (λex = 370 nm; λem = 492 nm).

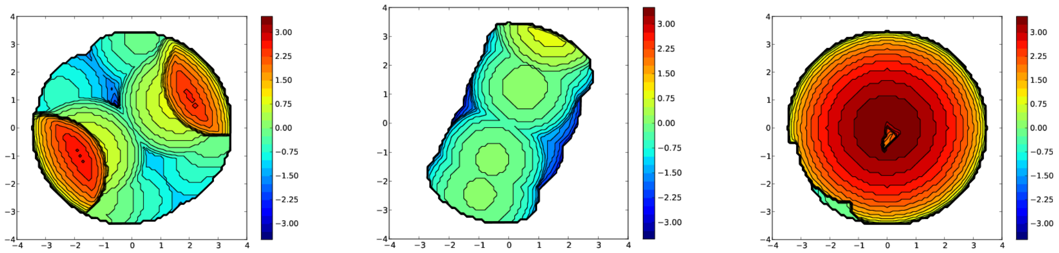

Figure S19. Topographical steric maps for xantphos, Htbz and {(xantphos)+(Htbz)} for compound **1**.

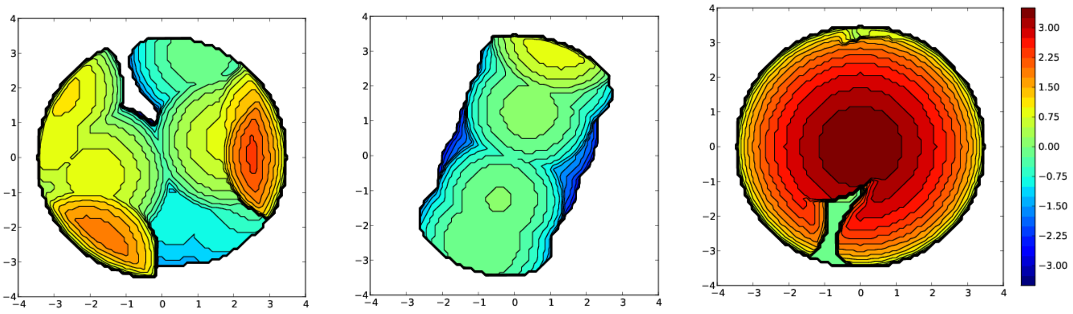

Figure S20. Topographical steric maps for dpephos, Htbz and {(dpephos)+(Htbz)} for compound **2**.

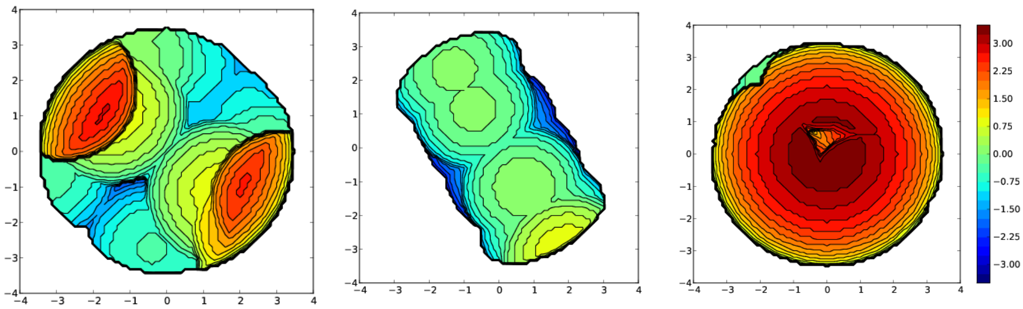

Figure S21. Topographical steric maps for xantphos, tbz$^-$ and {(xantphos)+(tbz$^-$)} for compound **3**.

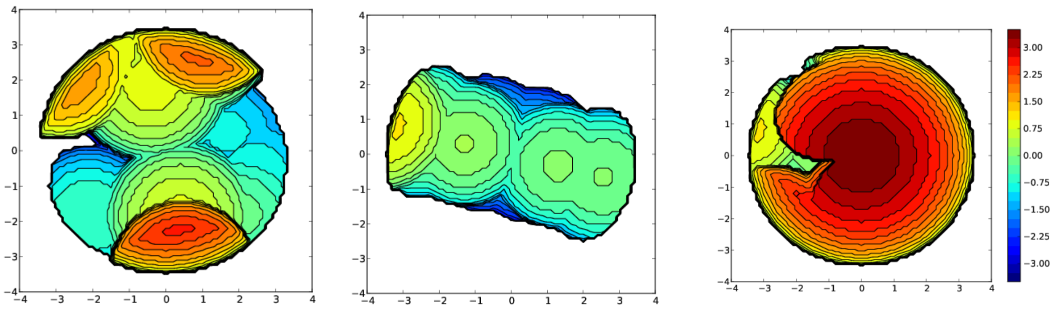

Figure S22. Topographical steric maps for dpephos, tbz$^-$ and {(dpephos)+(tbz$^-$)} for compound **4**.

Table S1. %V_{bur} for P^P, N^N and {(P^P)+(N^N)} units in complexes **1**-**4** and [Cu(dppnc)(Htbz)]$^+$ from optimized structures. Values are smaller than those calculated from the crystal structure data but lower values of %V_{bur} are again found for the {(P^P)+(N^N)} and (P^P) units in [Cu(dppnc)(Htbz)].

**Compound**	**P^P[b]**	**N^N[b]**	**{(P^P)+(N^N)}**
**1**	53.9	30.5	84.3
**2**	54.2	30.6	84.7
**4**	54.0	31.7	85.3
**[Cu(dppnc)(Htbz)]**	49.9	31.3	81.0

Table S2. Quantum yields [Φ (%)] for different [Cu(P^P)(N^N)]$^{0/+}$ complexes

**Compound**	**N^ N**				
	**Htbz**	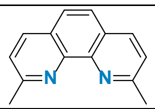			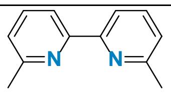
	**Air**	**Air**	**N_2[3]**	**O_2[3]**	**Air**
**[Cu(xantphos)(N^N)]$^+$**	1	11[2]	66	8.4	37[4]
**[Cu(dpephos)(N^N)]$^+$**	1	14[2]	88	19	43[5]
**[Cu(dppnc)(N^N)]**	10[1]	34[6]			27[6]

Table S3. Energy values for **1**, **2** and **4**

**Compound 1**	
Zero-point correction	0.752268 (Hartree/Particle)
Thermal correction to Energy	0.801986
Thermal correction to Enthalpy	0.802930
Thermal correction to Gibbs Free Energy	0.662923
Sum of electronic and zero-point Energies	-3405.072357
Sum of electronic and thermal Energies	-3405.022639
Sum of electronic and thermal Enthalpies	-3405.021694
Sum of electronic and thermal Free Energies	-3405.161701
**Compound 2**	
Zero-point correction	0.689142 (Hartree/Particle)
Thermal correction to Energy	0.736163
Thermal correction to Enthalpy	0.737107
Thermal correction to Gibbs Free Energy	0.600437
Sum of electronic and zero-point Energies	-3288.472072
Sum of electronic and thermal Energies	-3288.425050
Sum of electronic and thermal Enthalpies	-3288.424106
Sum of electronic and thermal Free Energies	-3288.560777
**Compound 4**	
Zero-point correction	0.675871 (Hartree/Particle)
Thermal correction to Energy	0.722424
Thermal correction to Enthalpy	0.723368
Thermal correction to Gibbs Free Energy	0.589755
Sum of electronic and zero-point Energies	-3288.046491
Sum of electronic and thermal Energies	-3287.999938
Sum of electronic and thermal Enthalpies	-3287.998994
Sum of electronic and thermal Free Energies	-3288.132606

References

[1]Alconchel, A.; Crespo, O.; García-Orduña, P.; Gimeno, M. C. *Closo*- or *Nido*-Carborane Diphosphane as Responsible for Strong Thermochromism or Thermally Activated Delayed Fluorescence (TADF) in [Cu(N^N)(P^P)]$^{0/+}$. *Inorg. Chem.* **2021**, *60*, 18521−18528.

[2]Li, C.; Mackenzie, C. F. R.; Said, S. A.; Pal, A. K.; Haghighatbin, M. A.; Babaei, A.; Sessolo, M.; Cordes, D. B.; Slawin, A. M. Z.; Kamer, P. C. J.; Bolink, H. J.; Hogan, C. F.; Zysman-Colman, E. Wide-Bite-Angle Diphosphine Ligands in Thermally Activated Delayed Fluorescent Copper(I) Complexes: Impact on the Performance of Electroluminescence Applications. *Inorg. Chem.* **2021**, *60*, 10323-10339.

[3]Smith, C. S.; Branham, C. W.; Marquardt, B. J.; Mann, K. R. Oxygen Gas Sensing by Luminescence Quenching in Crystals of Cu(xantphos)(Phen)$^+$ Complexes. *J. Am. Chem. Soc.* **2010**, *132*, 14079-14085

[4]Keller, S.; Pertegas, A.; Longo, G.; Martínez, L.; Cerdá, J.; Junquera-Hernández, J. M.; Prescimone, A.; Constable, E. C.; Housecroft, C. E.; Orti, E.; Bolink, H. J. Shine Bright or live long: substituent effects in [Cu(N^N)(P^P)]$^+$ -based light-emitting electrochemical cells where N^N is a 6-substituted 2,2′-bipyridine. *J. Mater. Chem. C* **2016,** *4*, 3857-3871.

[5]Keller, S.; Constable, E. C.; Housecroft, C. E.; Neuburger, M.; Prescimone, A.; Longo, G.; pertegas, A.; Sessolo, M.; Bolink, H. J. [Cu(bpy)(P^P)]+ containing light-emitting electrochemical cells: improving performace through simple substitution. *Dalton Trans.* **2014**, *43*, 16593-16596.

[6]Cheng, G.; So, G. K.-M.; To, W.-P.; Chen, Y.; Kwok, C.-C.; Ma, C.; Guan, X.; Chang, X.; Kwok, W.-M.; Che. C.-M. Luminescent Zinc(II) and Copper(I) Complexes for High-Performance Solution Processed Monochromic and White Organic Light-Emitting Devices. *Chem. Sci.* **2015**, *6*, 4623-4635.
